# Research highlights, *The Plant Genome*, Volume 16, Issue 1

**DOI:** 10.1002/tpg2.20322

**Published:** 2023-02-27

## 1 DROUGHT‐SMART FUTURE CROP PLANTS

Drought stress (DS) adversely affects agricultural productivity worldwide and is expected to rise in the coming years. Therefore, it is vital to understand the physiological, biochemical, and molecular mechanisms associated with DS. Raza et al. (https://doi.org/10.1002/tpg2.20279) examined recent advances in plant responses to DS to expand our understanding of DS‐associated mechanisms. To cope with DS, we recommend incorporating several approaches, such as multi‐omics (genomics, transcriptomics, proteomics, metabolomics, and epigenomics), genome editing, conventional and speed breeding, phytohormone treatments, and agronomic platforms, to develop drought‐smart cultivars to achieve the ‘zero hunger’ goal.

## 2 SOYBEAN UBIQUITIN–PROTEASOME SYSTEM

Increasing soybean yield has become a worldwide scientific problem in the world. Xiong et al. (https://doi.org/10.1002/tpg2.20281) summarized current knowledge about soybean ubiquitin‐related proteins and outlined the method of combining phenotype, mutant library, transgenic system, genomics, and proteomics approaches to facilitate the exploration of soybean ubiquitin–proteasome system (UPS). The roles of soybean UPS in soybean improvements for yield and stress tolerance were discussed. This review will be helpful for soybean scientists to learn current research progress of soybean UPS and further lay a theoretical reference for molecular improvement of soybean in the future.

## 3 COMPARING ARTIFICIAL INTELLIGENCE TECHNIQUES WITH THE STATE‐OF‐THE‐ART PARAMETRIC PREDICTION MODELS FOR PREDICTING SOYBEAN TRAITS

Soybean is one of the major crops in agriculture, and one of the major tasks in soybean breeding is to develop superior lines in terms of yield, protein, and oil content. Genomic prediction is a technique where genotypic and phenotypic information are used to predict the performance of unobserved lines in fields to aid the selection process. In this study, Ray et al. (https://doi.org/10.1002/tpg2.20263) compared the state‐of‐the‐art parametric genomic prediction models with nonparametric artificial intelligence–based techniques in terms of prediction accuracy. Here, prediction accuracy is defined as the correlation between the true (or observed) and the predicted phenotypic values within the same environment. They concluded that in general the conventional model performed the best when the genotypic and environmental main effects, and the genotype‐by‐environment interaction were formally included into the model. Moreover, their conclusions are agreed with other similar studies showing that the performance of the artificial intelligence–based models is highly influenced by the tuning of the hyperparameters and the size of the training data; thus, further investigation is necessary.

## 4 UNDERSTANDING FLOWERING TIME BETTER USING NOVEL PHENOTYPES

Improved understanding of the genetics underlies when plants’ flower is key to broadening genetic diversity and overcomes adaptation constraints in plant‐breeding programs. Neupane et al. (https://doi.org/10.1002/tpg2.20269) took flowering time and environmental data from a trial of diverse lentil germplasm grown in multiple locations around the world to reveal genes associated with this trait. To do this, they derived latent variable phenotypes from mathematical models for use as main traits and covariates. Regions of the genome associated with response to temperature and photoperiod were identified that reinforced the role of known genes and pointed to previously unknown associations. Their approach can be replicated with other crop species for understanding complex traits such as flowering time.

## 5 TARGETED GENOTYPING DEVELOPED FOR SOYBEANS

Enabling genomic selection for soybean progeny rows requires a genome‐wide genotyping method that provides a low per‐sample cost. Molecular inversion probe (MIP) is a targeted genotyping method that is low cost and can provide high‐quality data. Wang et al. (https://doi.org/10.1002/tpg2.20270) reported developing a 1K MIP SNP set with markers selected to maximize genetic polymorphism when used to screen soybean‐breeding populations. The MIP set was found to provide accurate genotyping and coverage across the genome in elite soybean populations. MIPs will be a powerful tool that will enable genomic selection within soybean‐breeding programs.

## 6 *Phytophthora* RESISTANCE IN DIVERSE STRAWBERRIES

*Phytophthora cactorum* causes crown rot, a devastating disease of global importance in cultivated strawberry. Jiménez et al. (https://doi.org/10.1002/tpg2.20275) reported a single large‐effect locus, RPc2, contributing to genetic resistance to *Phytophthora* crown rot in strawberry. RPc2 alone accounts for up to 50% of the genetic variance and improves genomic prediction model accuracy by up to 15% when included as a fixed effect in diverse strawberry germplasm. Although complete genetic resistance is rare, the beneficial allele of RPc2 is present in all modern University of California—Davis germplasm. Thus, marker‐assisted and genomic selection incorporating RPc2 may be robust solutions for improving and identifying donors of genetic resistance to *Phytophthora* crown rot in strawberry.

## 7 LIGNIN INTERWOVEN IN MAIZE DEFENSE

Lignin plays an integral part in plant structure, function, and defense. In maize, brown midrib (*bm*) mutants define a desirable market class of silage based on lower lignin levels that result increased digestibility in ruminant livestock. Kolkman et al. (https://doi.org/10.1002/tpg2.20278) showed that *bm* mutants (*bm1*–*bm4*) are also more susceptible to foliar fungal (northern leaf blight [NLB], gray leaf spot, and anthracnose leaf blight) and bacterial (Stewart's wilt) diseases, and in some cases susceptible to *Gibberella* ear rot and anthracnose stalk rot. We examine the previously described quantitative trait loci (QTLs) and correlations between disease traits and stalk strength, as a proxy for lignin, to determine if there is evidence for pleiotropy. More specifically, a high resolution genome‐wide association study for resistance to NLB revealed candidate genes for resistance to NLB with diverse functions, including lignin‐ and nonlignin‐related genes. Lignin‐related genes ranged from developmental genes, transcription factors, monolignol, and monolignol processing genes. Breeding for resistance in a market class defined by a biochemical pathway, such as low lignin silage maize, can be achieved through selection of resistance genes from nontarget pathways.

## 8 GENOMIC SELECTION IN TEA BREEDING

Tea‐breeding programs traditionally use phenotype information to identify the best individuals as parents of next generations or as potential new varieties evaluated in extensive field trials. This phenotype‐based selection has a limited accuracy and is also a time‐consuming—it takes about 16 years to develop a new variety for commercial release. Genomic selection first trains a prediction model on a population of genotyped and phenotyped individuals and then uses that model to predict breeding values of selection candidates early in a breeding cycle using only genome data. Lubanga et al. (https://doi.org/10.1002/tpg2.20282) showed how to leverage genomic selection in tea breeding with two competitive approaches. When applied at the early seedling stage, genomic selection is predicted to generate 1.7 times more genetic gain than the traditional phenotype‐based selection using the same budget.

## 9 CARBON 13 RATIO PLASTICITY IDENTIFIED IN SOYBEAN

Water use efficiency (WUE) is potentially an important trait for improving soybean drought tolerance, but WUE is difficult to determine in field experiments and increased WUE may decrease growth and yield when soil moisture is not limiting. The ability to change WUE, or C13 ratio, in response to the environment is called plasticity and could potentially overcome the problem of low yields in favorable environments for genotypes with high WUE. Chamarthi et al. (https://doi.org/10.1002/tpg2.20284) used the ratio of C13 to C12 (C13 ratio) in plant tissue as a surrogate measure of WUE and evaluated 473 accessions for C13 ratio in several diverse environments. Chamarthi et al. identified accessions that were highly plastic for C13 ratio and used association mapping to identify chromosomal regions closely associated with C13 ratio and with plasticity. These results represent a genetic resource for improving soybean drought tolerance.

## 10 CHARACTERIZATION OF *Rsg3*, A NOVEL GREENBUG RESISTANCE GENE FROM THE CHINESE BARLEY LANDRACE PI 565676

Greenbug (*Schizaphis graminum* Rondani) is a pest that poses a serious threat to cereal production worldwide, and resistance genes are urgently needed to enhance greenbug resistance in barley. Xu et al. (https://doi.org/10.1002/tpg2.20287) discovered a novel greenbug resistance gene, *Rsg3*, in the Chinese landrace PI 565676 and mapped *Rsg3* to an interval of <100 kb, in which four genes were annotated. An allelism test indicated that *Rsg3* is independent of the *Rsg1* locus, with an estimated recombination frequency of 12.85% ± 0.20% and a genetic distance of 13.14 ± 0.21 cM between the two loci. Two SNPs flanking *Rsg3* were converted to Kompetitive Allele Specific PCR markers, which can be used to tag *Rsg3* in barley breeding.

## 11 ARABIDOPSIS DUF239 FAMILY

The domain of unknown function 239 (DUF239) is found in Neprosins, which are glutamic‐proteases specific to carnivorous plants of the *Nepenthes* genus. The existence of DUF239 sequences in other plant species is poorly documented. Vergès et al. (https://doi.org/10.1002/tpg2.20290) provided the inventory, phylogeny, and domain organization of 50 DUF239 in Arabidopsis. Most of DUF239 genes are expressed in seed endosperm and their domain organization resembles that of Neprosins. Expression pattern analyzes and the presence/absence of specific Glu/Gln dyads related to glutamic‐protease catalytic cycle suggest a new role of the DUF239 proteins during seed development.

## 12 THE MECHANISM OF HETEROSIS

Over the last century, geneticists have put forward several hypotheses, including dominance, overdominance, and epistasis, to explain heterosis. But the mechanism of heterosis is still unclear. Wang et al. (https://doi.org/10.1002/tpg2.20293) found the immature ear length of hybrid at the IM stage fitted an additive (midparental) model, which showed high parental dominance at the spikelet‐pair meristem stage. The distinct maternal or paternal genes (genes expressed only in one parental line and their hybrid but silenced in another line) lead to a richer variety of genes of hybrids than parental lines and a nonadditive expression pattern. Nonadditive expression made the hybrid show stronger vigor than both parents. Cell redox homeostasis genes that maintain overdominant expression may have an important contribution to heterosis.

## 13 META‐QTL ANALYSIS FOR CHLOROPHYLL TRAITS OF WHEAT

Guo et al. (https://doi.org/10.1002/tpg2.20294) identified a total of 56 meta‐QTLs (MQTLs) for chlorophyll traits by QTL meta‐analysis, more than half of which were validated by marker–trait associations in genome‐wide associations studies. Six MQTLs were selected as breeders’ MQTLs. In addition, they have identified 21 candidate genes that are significantly enriched in chlorophyll metabolism and photosynthetic pathways, mainly related to chlorophyll metabolizing enzymes and photosynthetic proteins.

## 14 SCREENING OF HD‐ZIP GENES RELATED TO PRICKLE DEVELOPMENT IN *Zanthoxylum armatum*

Plant prickles can play a protective role. On the other hand, they cause a lot of trouble in production. Zhang et al. (https://doi.org/10.1002/tpg2.20295) identified 76 homeodomain leucine zipper (HD‐ZIP) genes from the genome of *Zanthoxylum armatum* and divided them into four subfamilies (I–IV) based on phylogenetic analysis, conservative motif composition, and gene structure analysis. In addition, the chromosome location, synteny, and *cis*‐elements of *ZaHDZ* gene were analyzed. Among all *ZaHDZ* genes, the *ZaHDZ16* gene was specifically highly expressed in the prickles and was found to be highly homologous to some HD‐ZIP genes related to trichome development by sequence comparison, thus hypothesizing that *ZaHDZ16* may be involved in the formation of prickles *Z. armatum*.

## 15 SEQUENCING WHEAT REGULATORY REGIONS

Regulatory regions of the genome contain variation that is valuable for crop improvement. Zhang et al. (https://doi.org/10.1002/tpg2.20296) developed a new target enrichment assay that facilitates cost‐effective sequencing of the regulatory regions of the large wheat genome. This assay includes the promoters of all high‐confidence‐annotated wheat genes and regions of open chromatin enriched for regulatory elements. Test captures on hexaploid and tetraploid wheat and other diploid cereals show that the assay has broad potential utility for cost‐effective promoter and open chromatin resequencing and general‐purpose genotyping of various *Triticeae* species.

## 16 GENOMICS‐GUIDED SELECTION OF CHIPPING POTATO CLONES

Genomic selection can speed up the plant breeding process by quickly identifying the best parents and selecting the most promising progenies. Pandey et al. (https://doi.org/10.1002/tpg2.20297) used genomics‐guided selection based on multi‐trait selection indexes to identify potato clones with light‐color chips, good chip quality, high yield, and high specific gravity. The selected clones can be used as parents or advanced toward developing new chipping potato varieties. The quantitative genetic nature of important traits such as vine maturity and chip color was confirmed, and QTLs were mapped. This research provides valuable insights into the genetics of chip‐related traits and guides selection to develop new chipping varieties faster and more efficiently.

## 17 GWAS UNCOVERS GENOMIC REGIONS WITH POTENTIAL TO ENHANCE WHEAT YIELD

Wheat yield is a complex trait, which is primarily determined by different spike and kernel‐related traits. Understanding the genetic architecture underlying these key traits can help develop high‐yielding wheat cultivars. Halder et al. (https://doi.org/10.1002/tpg2.20300) identified several important genomic regions associated with important spike and kernel‐related traits in hard winter wheat using genome‐wide association analysis. The study also identified several wheat genotypes having different combinations of superior alleles that can directly be utilized for breeding novel high‐yielding winter wheat cultivars using marker‐assisted selection.

